# Supplementary figures and images for: Deciphering the Olfactory Mechanisms of Sitotroga cerealella Olivier (Lepidoptera: Gelechiidae): Insights from Transcriptome Analysis and Molecular Docking
Source: Insects. 2025 Apr 27;16(5):461. doi: 10.3390/insects16050461 (PMC12112289; doi:10.3390/insects16050461)

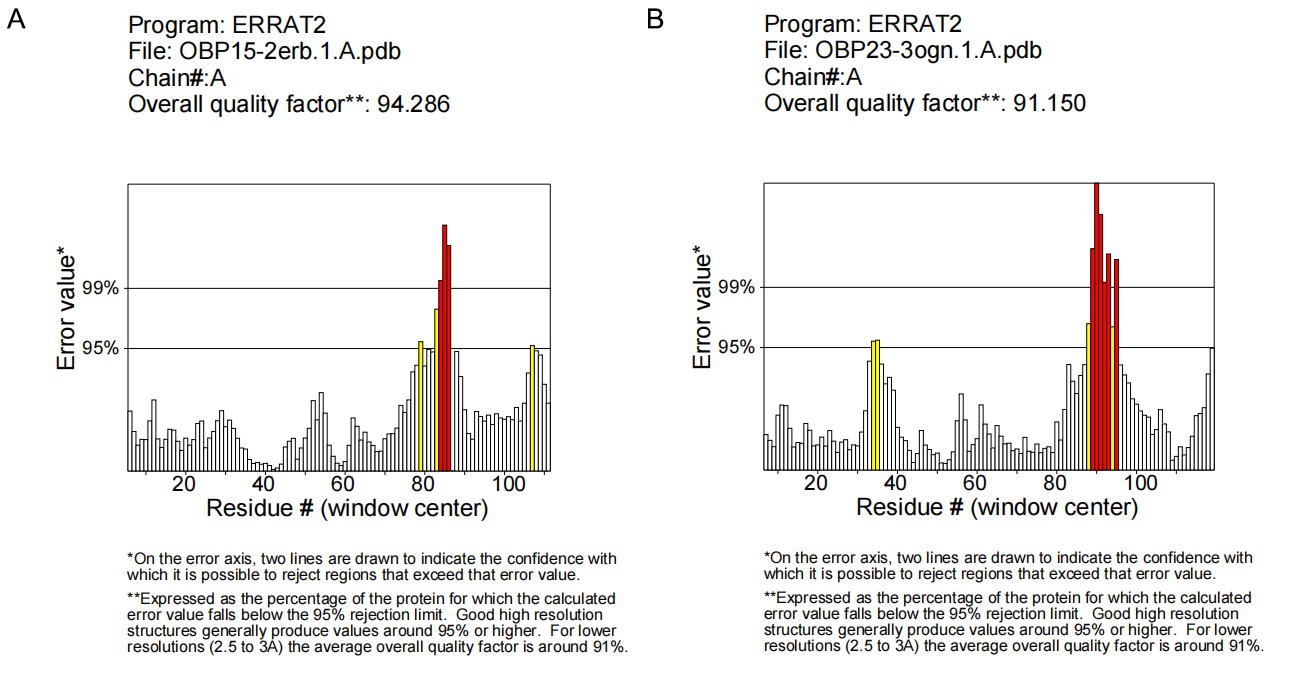

Supplement: Supplementary file 1 [file insects-16-00461-s001.zip › Figure S1 Error value of ERRAT calculation of modeled ScerOBP15 and ScerOBP23.tif]

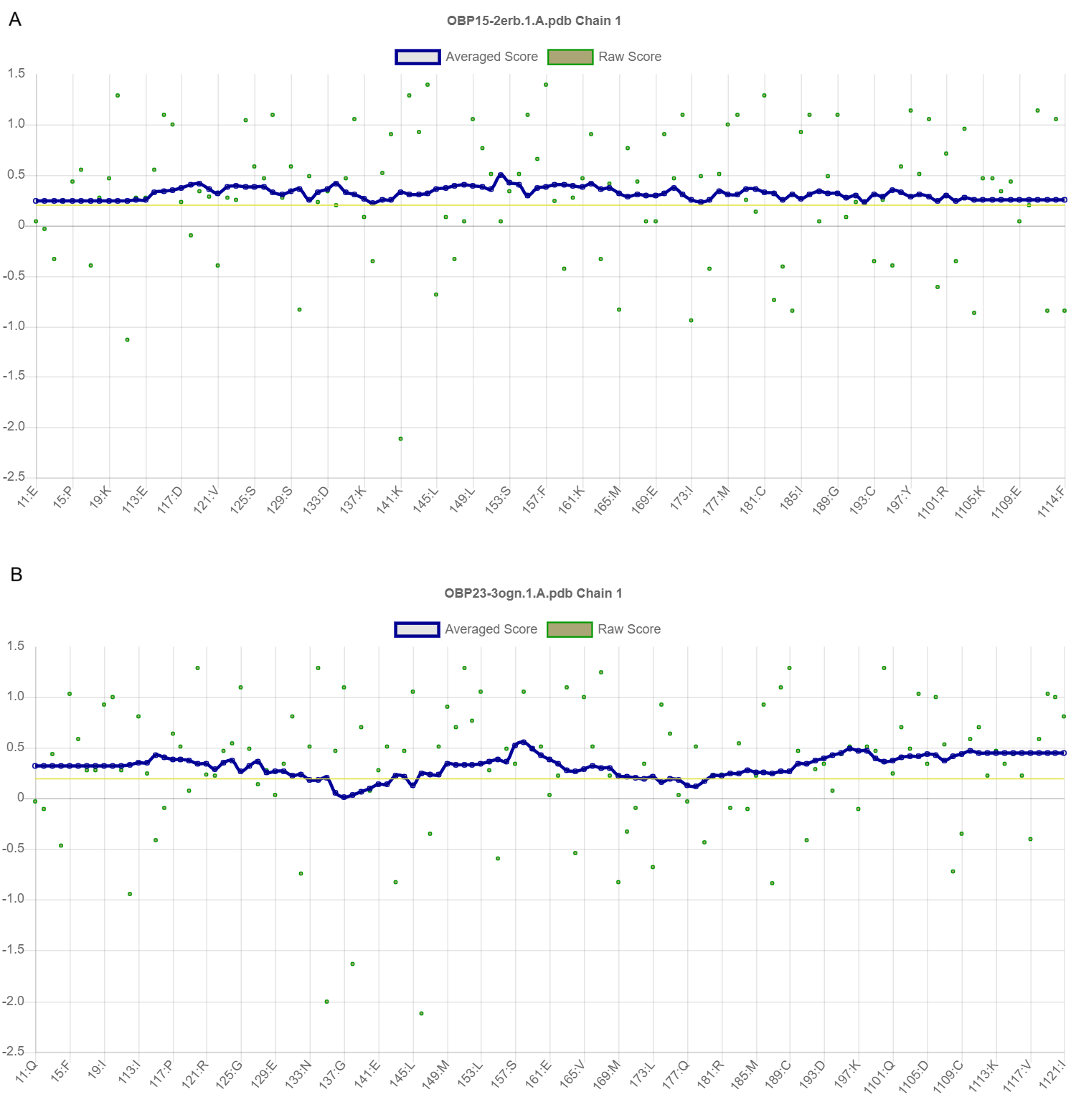

Supplement: Supplementary file 1 [file insects-16-00461-s001.zip › Figure S2 Verify-3D evaluation of the model of ScerOBP15 and ScerOBP23.tif]

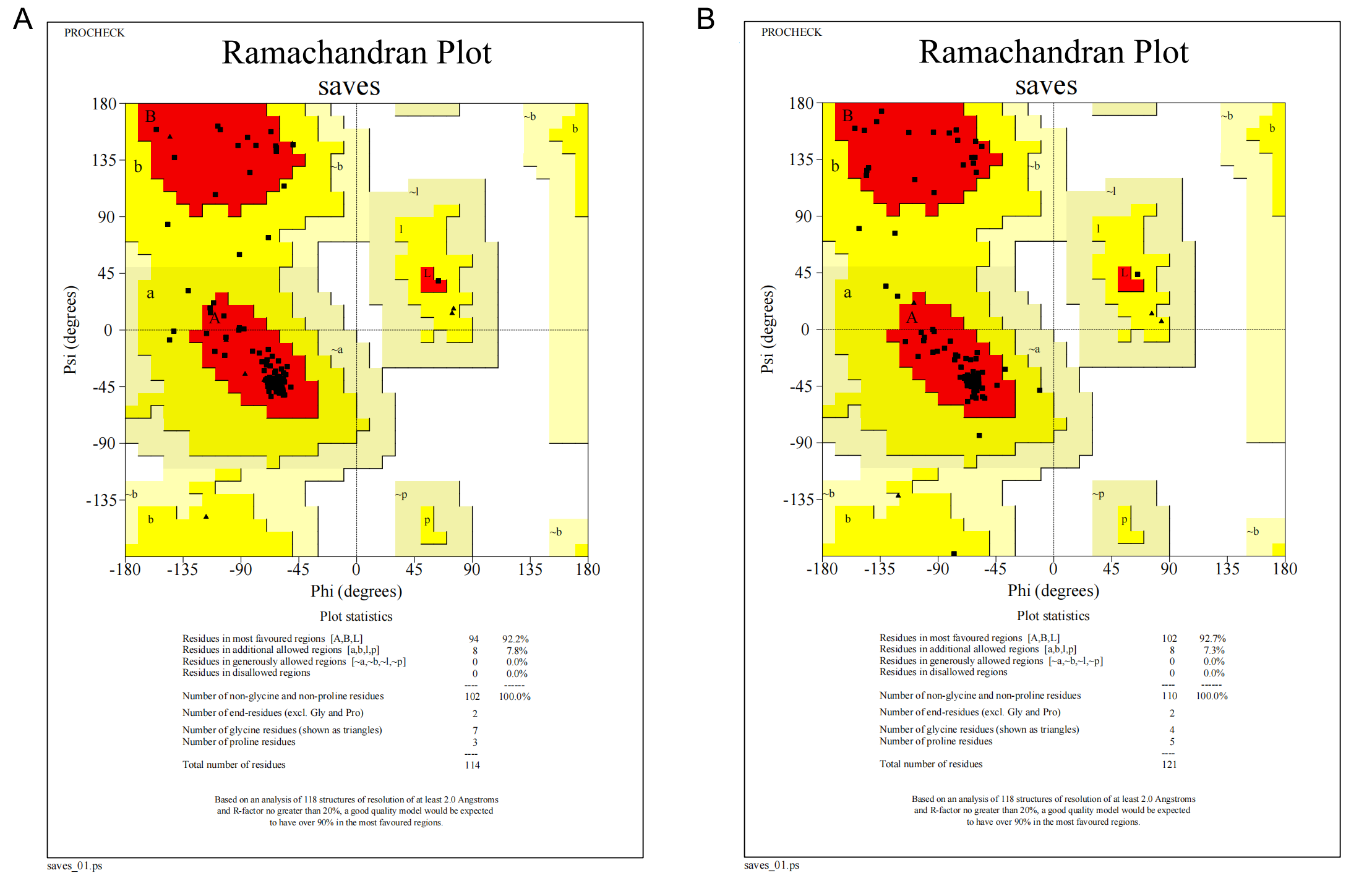

Supplement: Supplementary file 1 [file insects-16-00461-s001.zip › Figure S3 Procheck's evaluation of ScerOBP15 and ScerOBP23 model.tif]

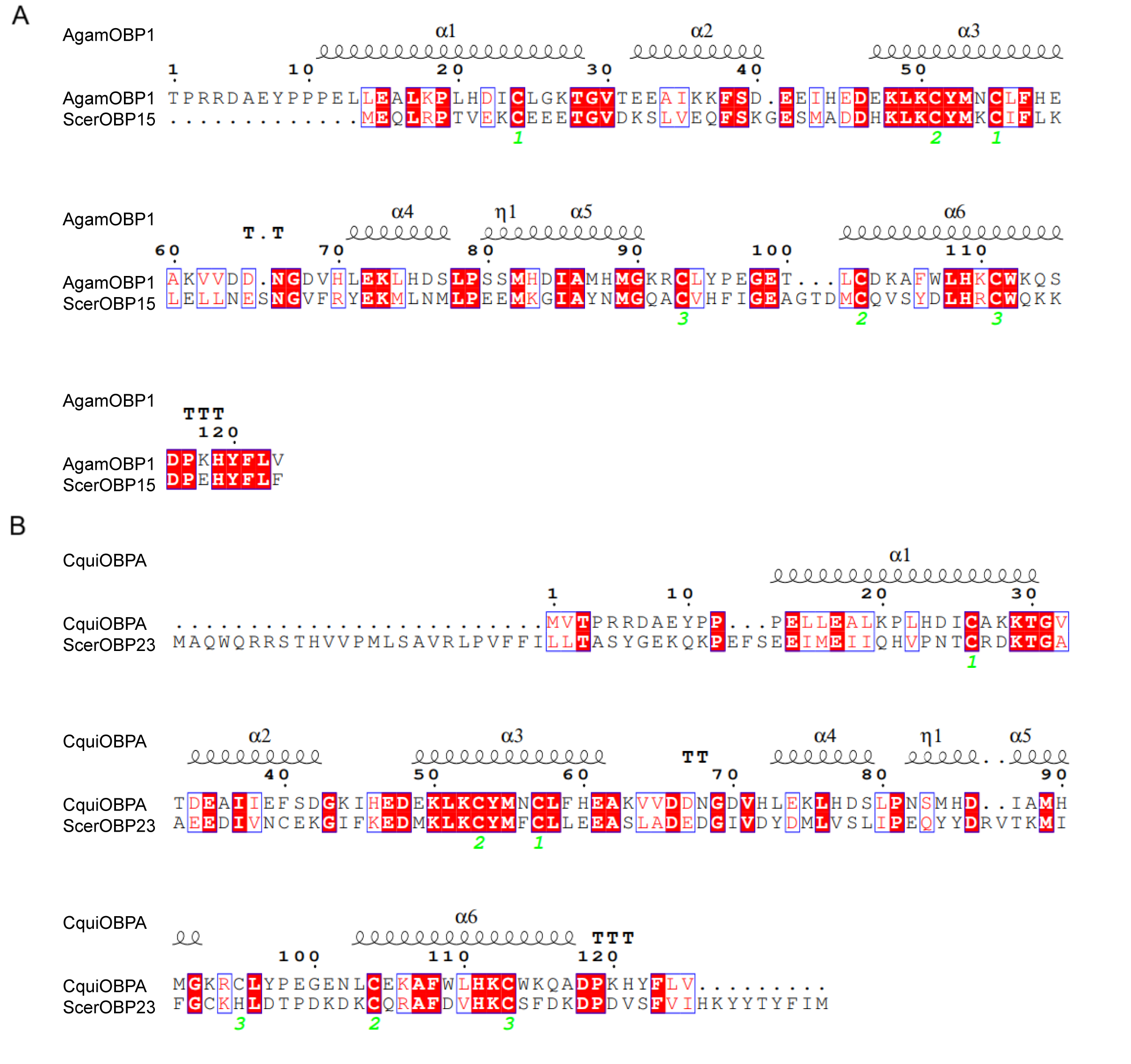

Supplement: Supplementary file 1 [file insects-16-00461-s001.zip › Figure S4 Sequence alignment of ScerOBP15 and ScerOBP23 with homology modeling templates.tif]
